# TAOK3 Facilitates Esophageal Squamous Cell Carcinoma Progression and Cisplatin Resistance Through Augmenting Autophagy Mediated by IRGM

**DOI:** 10.1002/advs.202300864

**Published:** 2023-09-13

**Authors:** Mingchuang Sun, Zhaoxing Li, Xiaoyuan Wang, Meirong Zhao, Yuan Chu, Zehua Zhang, Kang Fang, Ziying Zhao, Anqi Feng, Zhuyun Leng, Jianing Shi, Li Zhang, Tao Chen, Meidong Xu

**Affiliations:** ^1^ Endoscopy Center Department of Gastroenterology Shanghai East Hospital School of Medicine Tongji University Shanghai 200120 China; ^2^ Shanghai East Hospital Jinzhou Medical University Liaoning 121001 China; ^3^ Department of Pathology Shanghai East Hospital School of Medicine Tongji University Shanghai 200120 China

**Keywords:** Autophagy, Chemoresistance, Esophageal squamous cell carcinoma, IRGM, KMT2C, TAOK3

## Abstract

Esophageal squamous cell carcinoma (ESCC) is one of the deadliest cancers because of its robust aggressive phenotype and chemoresistance. TAO kinase belongs to mitogen‐activated protein kinases, which mediate drug resistance in multiple cancers. However, the role of TAO kinase in ESCC progression and chemoresistance has never been explored. Here, it is reported that TAOK3 augments cell autophagy and further promotes ESCC progression and chemoresistance. Mechanistically, TAOK3 phosphorylates KMT2C at S4588 and strengthens the interaction between KMT2C and ETV5. Consequently, the nuclear translocation of KMT2C is increased, and the transcription of autophagy‐relevant gene IRGM is further upregulated. Additionally, the inhibitor SBI‐581 can significantly suppress cell autophagy mediated by TAOK3 and synergizes with cisplatin to treat ESCC in vitro and in vivo.

## Graphical abstract

TAOK3 can augment autophagy and chemo‐resistance in esophageal squamous cell carcinoma (ESCC) cells mechanically by phosphorylating KMT2C and promoting the interaction between KMT2C and ETV5. Consequently, the nuclear translocation of KMT2C is increased, and IRGM transcription is promoted. Particularly, a specific inhibitor of TAOK3, SBI‐581, is identified, which can synergize with cisplatin and subsequently enhance the effectiveness of chemotherapy in ESCC.

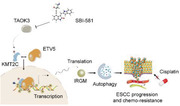

## 1 Introduction

Esophageal cancer ranks sixth in mortality in human cancer, among which c (ESCC) accounts for approximately 90%.^[^ ^1^ ^]^ ESCC is related to the extensive lymphatic spread and is always diagnosed at advanced stages.^[^ ^2^, ^3^, ^4^ ^]^ Chemotherapy is crucial for ESCC patients, as it plays an irreplaceable role in postoperative adjuvant therapy and first‐line treatment.^[^ ^5^ ^]^ Cisplatin is still the foremost agent for treating ESCC, but drug resistance limits its actual effect and worsens ESCC patients’ prognosis.^[^ ^6^ ^]^ Thus, it is of great significance to figure out the underlying mechanism of tumorigenesis and cisplatin resistance of ESCC and discover novel therapeutic targets and promising prognostic markers of ESCC.

Thousand‐and‐one (TAOK) kinases are serine/threonine kinases and belong to the SET20 kinase family.^[^ ^7^ ^]^ As a MAP3K, TAO kinase reacts to stimuli, such as ultraviolet irradiation, cytokines, heat, nutrition deficiency, and osmotic shock.^[^ ^8^ ^]^ Under ionizing and ultraviolet radiation simulation, TAO kinase was reported to be phosphorylated and activated by ATM (ataxia telangiectasia mutated) and further regulate p38‐mediated DNA damage responses.^[^ ^9^ ^]^ Additionally, apart from MKK3/6 in the p38 MAPK cascade, TAO kinases were found to phosphorylate MKK4/7 and activate the JNK signaling cascade.^[^ ^10^, ^11^, ^12^ ^]^ The Hippo signaling pathway, also known as Salvador–Warts–Hippo (SWH) pathway, regulates cell proliferation and apoptosis.^[^ ^13^ ^]^ TAO kinases have also been found to regulate Hippo signaling pathway, in addition to their involvement in the MAPK cascades.^[^ ^14^ ^]^ Apart from regulating various signaling pathways, TAO kinases are verified to interact with other cellular proteins, both dependent and independent of their kinase activity. Through interacting with some target proteins, TAO kinases regulate the DNA damage responses, cytoskeleton stability, apoptosis, and other physiological and pathophysiological responses.^[^ ^8^ ^]^ It is noteworthy that TAO kinases were reported to mediate chemotherapy resistance in many cancers.^[^ ^15^, ^16^ ^]^ Yet, the role of TAO kinases in ESCC has never been explored.

In this study, autophagy generally referring to “macroautophagy” is a common phenomenon in cancer cells. In response to harsh conditions cancer cells face, cancer takes on eight hallmarks, and resistance to death is one of them.^[^ ^17^ ^]^ As a main method of resistance to death, autophagy helps cancers recycle catabolites and use them for biosynthesis and energy generation. Besides, apart from drug efflux and metabolism, tumor heterogeneity, inactivation of apoptosis, increased repair of damage, angiogenesis, mutation or loss of molecular targets, and compartmentalization, induction of autophagy is a vital approach by which cancer cells become resistant to chemotherapy.^[^ ^18^ ^]^ So far, many studies have reported that the autophagy level in ESCC is elevated,^[^ ^19^, ^20^, ^21^ ^]^ but its relationship with chemotherapy resistance and the clear mechanism needs to be further elucidated.

Here, among TAO kinases, we found only TAOK3 was upregulated and activated autophagy in ESCC cells and tissues. TAOK3 was associated with unfavorable clinic‐pathological characteristics and poor prognosis of ESCC patients. We showed that TAOK3 was required for tumorigenesis, metastasis, and cisplatin resistance of ESCC both in vitro and in vivo. Mechanistically, TAOK3 could phosphorylate KMT2C at S4588 and promote the interaction between KMT2C and ETV5 in nuclei, and the transcription of IRGM was increased consequently.

## 2 Results

### 2.1 TAOK3 of TAO Kinases Promotes Autophagy, Suppresses Apoptosis, and Further Augments Cisplatin Resistance In Vitro in ESCC

**Figure 1:**
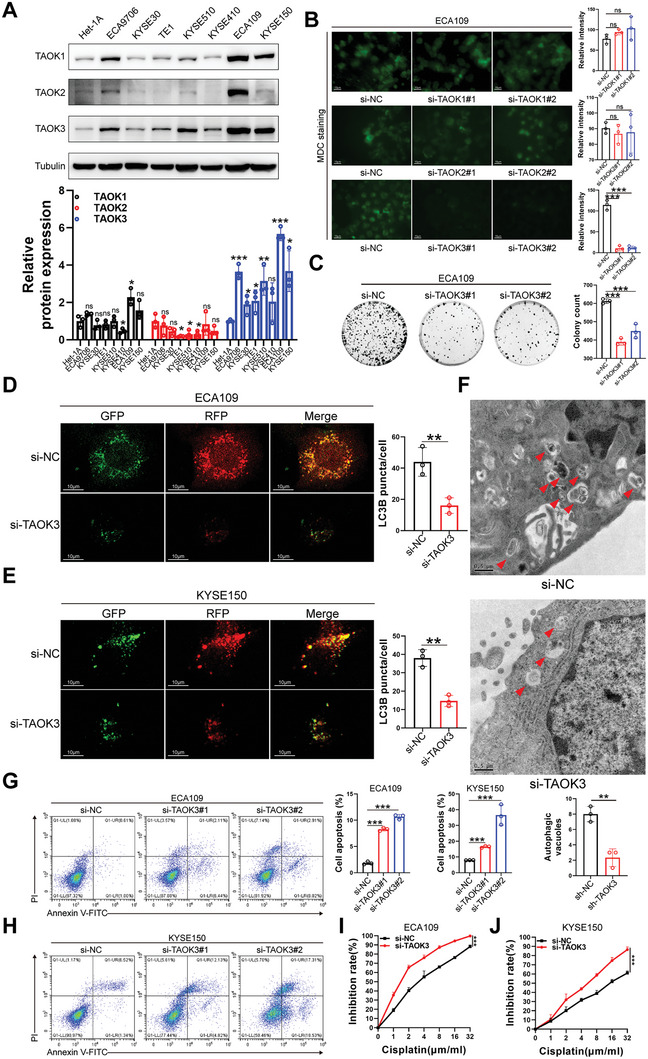
TAOK3 augments autophagy and cisplatin resistance but inhibits cell apoptosis in ESCC cells. A) The expression level of protein TAO kinases in ESCC cell lines. B) MDC staining analysis of acidic vesicular organelles formation when TAO kinases were knocked down. C) Colony formation assay determined the proliferation of ECA109 when TAOK3 was knocked down. D,E) Confocal microscopy analysis of autophagy‐lysosome formation when TAOK3 was knocked down after lentivirus vector carrying GFP‐RFP‐LC3 was transfected. F) TEM analysis of Autophagic vesicle formation when TAOK3 was knocked down in ECA109 cells. G,H) Annexin V‐FITC/PI staining analysis of cell apoptosis level when TAOK3 was knocked down in ESCC cells. I,J) CCK8 analysis of the inhibition rate of ESCC cells growth when TAOK3 was knocked down.

To investigate the association between TAO kinases and autophagy in ESCC, we first explored the expression level of TAO kinases in ESCC cell lines. We found that compared with normal esophageal epithelial cell Het‐1A, TAOK3 rather than TAOK1 or TAOK2 was significantly upregulated in ESCC cell lines (**Figure** **1**A). Besides, all these three kinases were relatively upregulated in cell lines ECA109 and KYSE150, so we chose these two cell lines to perform the following experiments. From TCGA analysis (Figure S1A–C, Supporting Information) and qPCR (Figure S1D–F, Supporting Information), and western blot (Figure S1G, Supporting Information) in ESCC tissues collected from five enrolled patients, we found that of these three TAO kinases, TAOK3 was confirmed to be overexpressed in ESCC, and its expression level in ESCC ranked high among all kinds of cancers (Figure S1H, Supporting Information). After all these three kinases were dramatically knocked down by siRNAs (Figure S1I–T, Supporting Information), we applied Monodansyl cadaverine (MDC) staining. Only TAOK3 of these three kinases was found to promote the formation of acidic vesicular organelles, including autophagic vacuoles (Figure 1B). Meanwhile, we found the proliferation of ECA109 cells was alleviated when TAOK3 was knocked down from colony formation assay (Figure 1C). After lentivirus vector carrying GFP‐RFP‐LC3 was transfected into ECA109 and KYSE150, we found when TAOK3 was knocked down, the formation of autophagy‐lysosome decreased significantly through confocal microscopy (Figure 1D,E). From Transmission electron microscopy (TEM) images, the reduction of Autophagic vesicles was also detected when TAOK3 was knocked down (Figure 1F). As we all know, autophagy can inhibit cell apoptosis in many cancers. After Annexin V‐FITC/PI staining assay was performed, we found knocking down of TAOK3 could significantly increase apoptosis of ESCC cells (Figure 1G,H). Furthermore, after TAOK3 was knocked down, we found the sensitivity of ESCC cells to cisplatin could be augmented (Figure 1I,J).

### 2.2 TAOK3 Promotes the Proliferation, Migration, and Invasion of ESCC Cells

The overexpression of TAOK3 by pcDNA3.1‐FLAG‐TAOK3 plasmid transfection was confirmed by qPCR and western blotting analysis (Figure S1U–X, Supporting Information). From colony formation and CCK8 assays of cell lines ECA109 and KYSE150 (Figure 1C and Figure S2A–G, Supporting Information), we found that TAOK3 overexpression could promote, but knockdown attenuated ESCC cells proliferation. To explore the alteration of the proliferation from the DNA duplication facet, we performed the Edu staining assay and found that the Edu incorporation was significantly decreased when TAOK3 was knocked down (Figure S2H–I, Supporting Information). The results of transwell assays indicated that both the migration and the invasion abilities of ECA109 and KYSE150 cells could be augmented when TAOK3 was overexpressed and alleviated when TAOK3 was knocked down (Figure S2J–Q, Supporting Information), which was consistent with the results of wound healing assay (Figure S2R–U, Supporting Information).

### 2.3 TAOK3 Phosphorylates KMT2C (at S4588), Strengthens the Interaction Between KMT2C and ETV5, and Further Promotes KMT2C Nuclear Translocation

**Figure 2:**
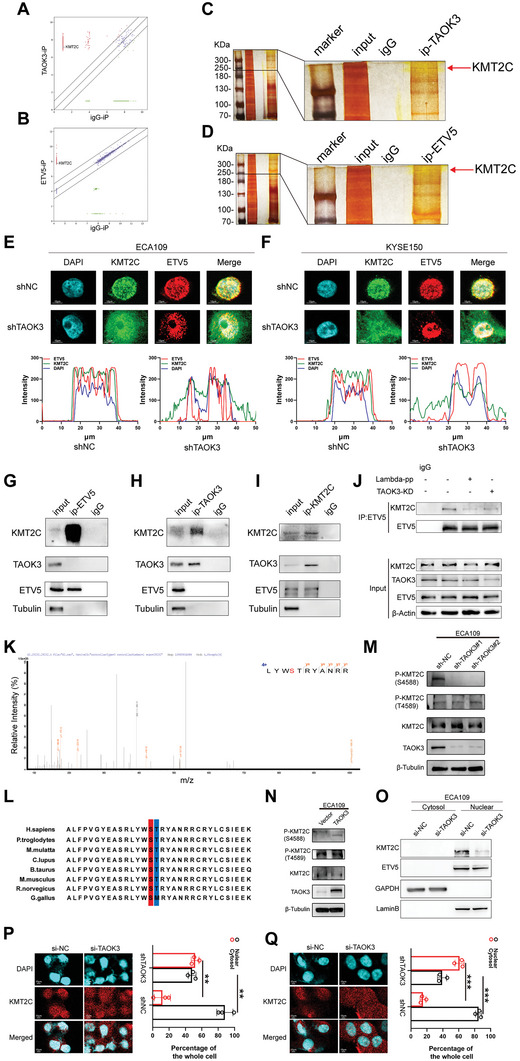
TAOK3 phosphorylates KMT2C (at S4588), strengthens the interaction between KMT2C and ETV5, and promotes KMT2C nuclear translocation. A,B) MS analysis of the potential binding proteins of TAOK3 and ETV5 in ECA109 cells. C,D) Silver staining assay showed bands of proteins immunoprecipitated by TAOK3 and ETV5 in ECA109 cells. E,F) IF analysis of the location of KMT2C and ETV5 in ESCC cells. G—I) Endogenous CO‐IP analysis of the interaction between TAOK3, KMT2C, and ETV5 in ECA109 cells. J) CO‐IP analysis of the interaction between KMT2C and ETV5 in ECA109 cells after L‐PP was used, or TAOK3 was knocked down. K) Phosphorylated KMT2C S4588 was identified by MS analysis. L) Sequence alignment around the S4588 and T4589 of KMT2C in various species. M) Western blot analysis of the expression of P‐KMT2C (S4588) and P‐KMT2C (T4589) when TAOK3 was knocked down in ECA109 cells. N) Western blot analysis of the expression of P‐KMT2C (S4588) and P‐KMT2C (T4589) when TAOK3 was overexpressed in ECA109 cells. O) Western blot analysis of nuclear and cytosol fractionated proteins in ECA109 cells. P,Q) IF analysis of the location of KMT2C in ESCC cells.

TAOK3 is a MAP kinase kinases (MAP3K) and it always works by phosphorylating some target proteins, so we performed mass spectrometry (MS) analysis to find the candidate interacted and phosphorylated proteins. The cutoff value was P<0.05 and FC >2. Intriguingly, after scanning the proteins bound by TAOK3, we surprisingly found that KMT2C also commonly called MLL3, which was ever captured by ETV5 in our previous study,^[^ ^22^ ^]^ ranked high among the enrolled proteins (**Figure** **2**A,B). ETS transcription factor family has been reported to influence cancer metabolism, especially autophagy in many studies.^[^ ^23^, ^24^, ^25^ ^]^ From the immunoprecipitation and silver staining results, we could easily find the band of protein KMT2C, which indicated that KMT2C might interact with TAOK3 and ETV5 respectively (Figure 2C,D). From Immunofluorescence (IF) assay, we found KMT2C and ETV5 were co‐localized in ESCC cells, especially in nuclei. However, the interaction and co‐localization could be released when TAOK3 was knocked down (Figure 2E,F). The further endogenous Co‐immunoprecipitation (CO‐IP) assay validated the interaction between endogenous TAOK3 and KMT2C (Figure 2G,I) and the interaction between endogenous ETV5 with KMT2C (Figure 2H,I). However, there was no interaction between TAOK3 and ETV5. After ECA109 cells were transiently transfected with Flag‐TAOK3 or Flag‐ETV5 constructs, we performed exogenous CO‐IP with anti‐Flag and confirmed the binding between protein TAOK3 and KMT2C and the binding between protein ETV5 and KMT2C in ECA109 cells (Figure S3A,B, Supporting Information). Because KMT2C is a histone H3K4 methyltransferase, it can synergistically work with transcription factor to affect the transcription of the downstream genes. Therefore, we proposed that TAOK3 might affect the interaction between KMT2C and ETV5 because of phosphorylating KMT2C. After ESCC cell lysate samples were treated with Lambda protein phosphatase (L‐pp), which could release phosphate groups from phosphorylated serine, threonine, and tyrosine residues in proteins, or cells treated with si‐TAOK3 or TAOK3 overexpression plasmid, CO‐IP was performed. We found that when L‐pp or si‐TAOK3 was used, the interaction between ETV5 and KMT2C was released. In contrast, when TAOK3 was overexpressed, the interaction between ETV5 and KMT2C was strengthened (Figure 2J and Figure S3C, Supporting Information). Also from the CO‐IP assay, when TAOK3 was knocked down, pan‐serine/threonine phosphorylated KMT2C was significantly downregulated (Figure S3D, Supporting Information). To further explore the specific phospho‐sites of KMT2C, we performed MS assay. The enriched phospho‐peptides were identified, and S4588 and T4589 were found to be the most potential phospho‐sites based on their ranking score (Figure S3E, Supporting Information and Figure 2K). In addition, the sequence alignment around S4588 and T4589 showed high conservation, with S4588 being more pronounced (Figure 2L). Moreover, we generated antibodies specifically recognizing P‐KMT2C (S4588) and P‐KMT2C (T4589) and examined KMT2C phosphorylation using a Western blot assay. We observed a significant downregulation of P‐KMT2C (S4588) expression when TAOK3 was knocked down (Figure 2M and Figure S3F, Supporting Information), and an upregulation when TAOK3 was overexpressed (Figure 2N and Figure S3G, Supporting Information). However, the change in P‐KMT2C (T4589) was not evident regardless of TAOK3 knockdown or overexpression. Western blot analysis of nuclear and cytosol fractionated proteins revealed that TAOK3 knockdown significantly decreased the expression of KMT2C in the nuclear protein fraction in ESCC cells (Figure 2O and Figure S3H, Supporting Information). IF staining confirmed nuclear accumulation of KMT2C decreased when TAOK3 was knocked down (Figure 2P,Q).

### 2.4 IRGM is the Target Gene of the TAOK3‐KMT2C Axis and is Transcriptionally Regulated by ETV5

**Figure 3:**
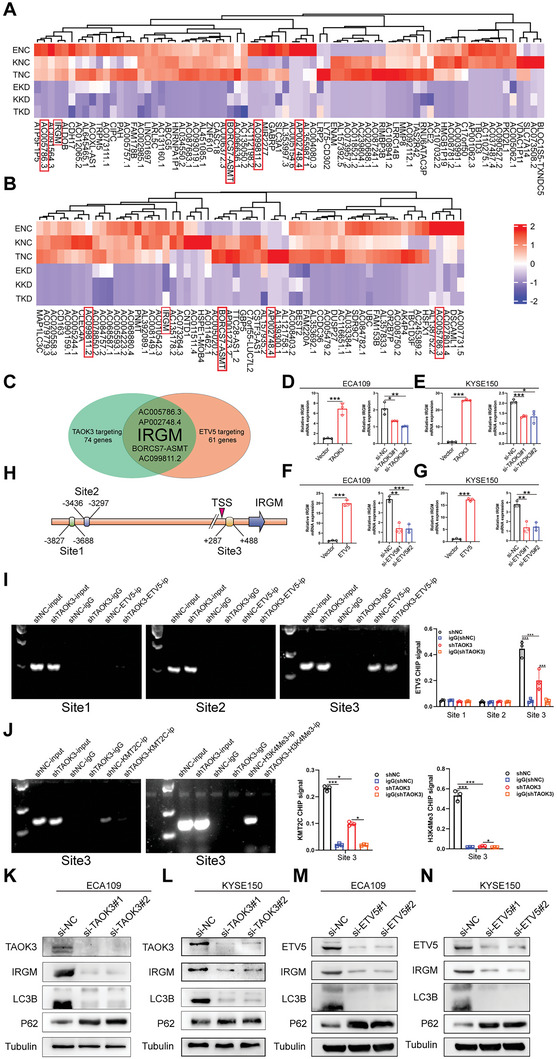
Gene IRGM is the downstream gene transcriptionally regulated by ETV5 and KMT2C. A,B) RNA‐seq analysis of the downstream genes regulated by TAOK3 and ETV5. C) The overlap of the genes regulated both by TAOK3 and ETV5 was shown. D,E) QPCR analysis of the IRGM mRNA expression level when TAOK3 was overexpressed or knocked down. F,G) QPCR analysis of the IRGM mRNA expression level when ETV5 was overexpressed or knocked down. H) The schematic diagram of the potential binding sites of ETV5 at the promoter of IRGM. I) CHIP‐qPCR analysis of the binding sites of ETV5 at IRGM promoter in ECA109 cells. J) CHIP‐qPCR analysis of whether KMT2C and H3K4Me3 bind to the promoter of IRGM in ECA109 cells. K–N) Western blot analysis of the expression of IRGM and autophagy‐associated proteins when TAOK3 or ETV5 was knocked down.

**Figure 4:**
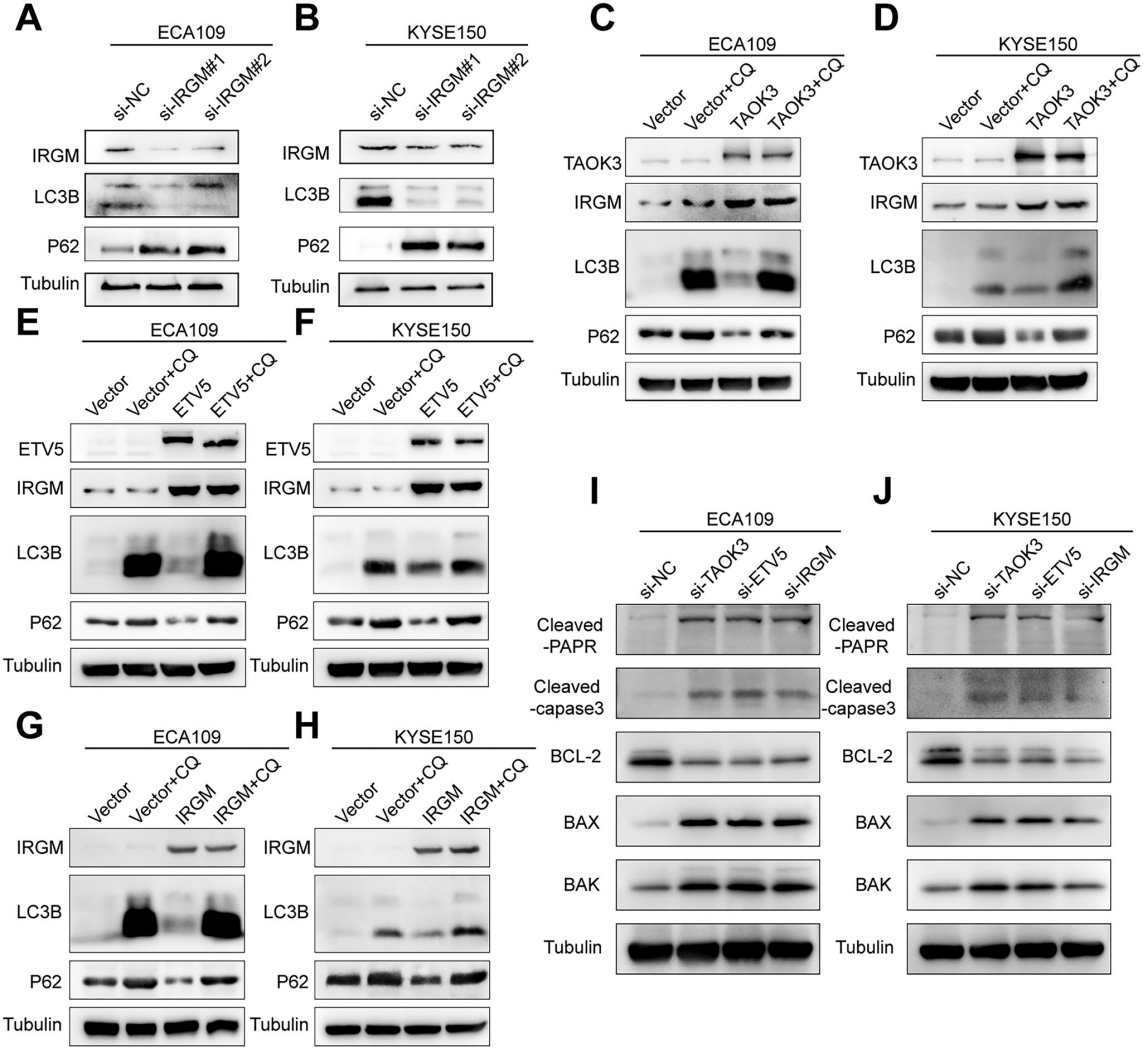
TAOK3, ETV5, and IRGM affect the expression of autophagy and apoptosis‐associated proteins. A,B) Western blot analysis of the expression level of autophagy‐associated proteins when IRGM was knocked down. C—H) Western blot analysis of the expression level of autophagy‐associated proteins when TAOK3, ETV5, and IRGM were overexpressed, or autophagy inhibitor CQ (25 umol L^−1^ for 24 h) was used. I—J) Western blot analysis of the expression level of apoptosis‐associated proteins when TAOK3, ETV5, and IRGM were knocked down.

To find the downstream genes regulated by TAOK3 and ETV5, we performed RNA‐seq assays when TAOK3 or ETV5 was knocked down or not in ECA109, KYSE150, and TE1 cell lines. The cutoff value is P<0.05 and FC>4 (**Figure** **3**A,B). After analyzing the alteration between si‐NC group and si‐TAOK3 group or si‐NC group and si‐ETV5 group, we overlapped the downregulated genes and found there were 5 genes enrolled (Figure 3C). Focusing on genes affecting autophagy in cancers, Immunity Related GTPase M (IRGM) attracted our attention. IRGM encodes a member of the p47 immunity‐related GTPase family. In addition to the involvement in the innate immune response, IRGM is well‐known for playing an essential role in regulating autophagy in many types of diseases, including cancers.^[^ ^26^, ^27^ ^]^ After the efficiency of ETV5 knockdown and overexpression was confirmed from qPCR (Figure S4A,C, Supporting Information) and western blot assays (Figure S4B,D, Supporting Information), we found that the expression of IRGM was significantly upregulated from the mRNA level when TAOK3 or ETV5 was overexpressed, and downregulated when TAOK3 or ETV5 was knocked down (Figure 3D–G, Supporting Information). To investigate whether IRGM is a direct ETV5 target gene, we conducted chromatin immunoprecipitation‐quantitative PCR (CHIP‒qPCR) assays to verify the three potential binding sites for ETV5 in the IRGM promoter predicted by Jaspar (Figure 3H). The CHIP assay showed a substantial increase in the binding of ETV5 to the chromatin region of the IRGM (site 3) promoter. Besides, when TAOK3 was knocked down, the binding was attenuated significantly (Figure 3I). Meanwhile, KMT2C and H3K4Me3 were also detected to bind this site and released when TAOK3 was knocked down (Figure 3J). From western blot assay, we found TAOK3 or ETV5 knockdown could downregulate the expression of protein IRGM and further downregulated the expression of LC3 II/I, but upregulated the expression of SQSTM1 (P62) protein (Figure 3K–N), and IRGM knockdown had the same effect (**Figure** **4**A,B). In contrast, TAOK3 or ETV5 overexpression had opposite effects (Figure 4C–F). Additionally, autophagy inhibitor chloroquine (CQ) could significantly reverse these effects (Figure 4C–F). Collectively, these data imply that under the effect of TAOK3's phosphorylation, KMT2C mediates histone methylation and transcriptionally upregulates the expression of IRGM together with ETV5, and tumor autophagy is augmented consequently. We further investigated the expression of apoptosis‐associated proteins. The results showed that TAOK3, ETV5, or IRGM knockdown could significantly decrease anti‐apoptosis protein BCL‐2 and increase apoptosis‐associated proteins BAK, BAX, cleaved caspase 3, and cleaved PARP (Figure 4I,J).

### 2.5 ETV5 and IRGM Promote Proliferation, Migration, Invasion, and Cisplatin Resistance in Vitro by Augmenting Autophagy

From the RT‐qPCR and western blot assays, we confirmed the overexpression and knockdown efficacy of IRGM (Figure S4E–H, Supporting Information). From the colony formation assays, we found that the overexpression of ETV5 or IRGM could augment ESCC cells proliferation, but knockdown is the opposite (Figure S4I–J, Supporting Information). These effects were further verified by CCK8 (Figure S4K–L, Supporting Information) and Edu staining assays (Figure S5A–D, Supporting Information). The results of transwell assays showed that the migration and invasion abilities increased when ETV5 or IRGM was overexpressed, but decreased when ETV5 or IRGM was knocked down (Figure S4M–P, Supporting Information), which was further validated from the wound healing assay (Figure S4Q–R, Supporting Information). The reduction of autophagic vesicles when ETV5 or IRGM was knocked down was observed from TEM (Figure S5E, Supporting Information). This effect was also detected from confocal images of ESCC cells transfected with lentivirus vector carrying GFP‐RFP‐LC3 (Figure S5F–I, Supporting Information). From the Annexin V‐FITC/PI staining assay, we found ETV5 or IRGM knockdown could significantly increase cell apoptosis (Figure S5J–K, Supporting Information). Furthermore, after ETV5 or IRGM was knocked down, we found the sensitivity of ESCC cells to cisplatin could be augmented (Figure S5L–M, Supporting Information).

### 2.6 The Specific TAOK3 Inhibitor SBI‐581 Could Help Suppress Proliferation, Inhibit Autophagy, Induce Cell Apoptosis, and Further Synergize with Cisplatin In Vitro

**Figure 5:**
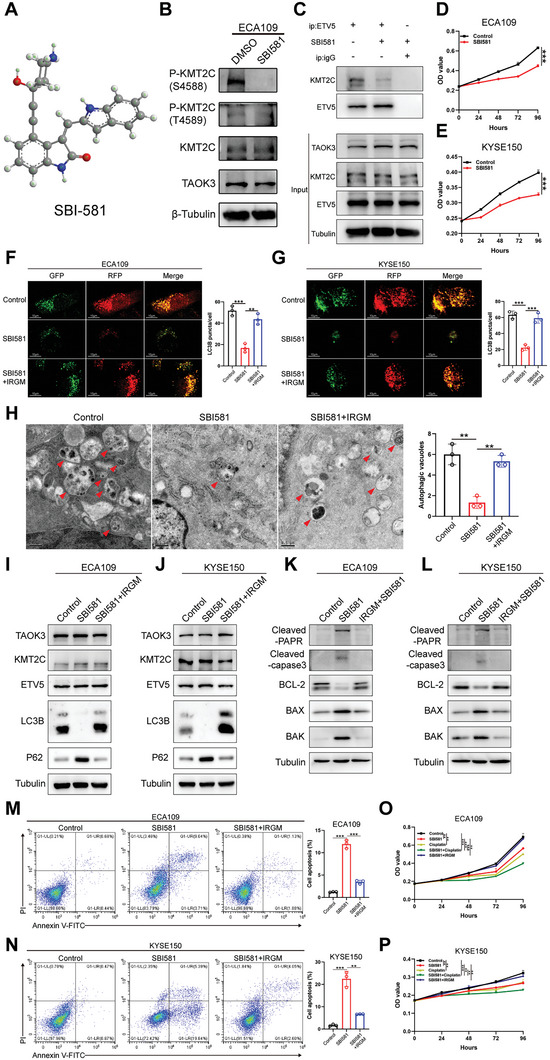
SBI‐581 inhibits the effect of TAOK3 in ESCC cells and synergizes with cisplatin in vitro. A) The molecular structure of SBI‐581. B) Western blot analysis of the expression of P‐KMT2C (S4588) and P‐KMT2C (T4589) when SBI‐581 was applied in ECA109 cells. C) CO‐IP analysis of the interaction between KMT2C and ETV5 when SBI‐581 (100 nm L^−1^) was used in ECA109 cells. D,E) CCK8 analysis of the viability of ESCC cells when SBI‐581 was applied. F,G) Confocal microscopy analysis of autophagy‐lysosome formation when ESCC cells treated with SBI‐581 or vector carrying IRGM plasmid. H) TEM analysis of Autophagic vesicle formation when SBI‐581 or vector carrying IRGM plasmid was used in ECA109 cells. I,J) Western blot analysis of the expression of autophagy‐related proteins when SBI‐581 or vector carrying IRGM plasmid was used. K,L) Western blot analysis of the expression of apoptosis‐related proteins when SBI‐581 or vector carrying IRGM plasmid was used. M,N) Annexin V‐FITC/PI staining analysis of cell apoptosis level when SBI‐581 or vector carrying IRGM plasmid was used in ESCC cells. O,P) CCK8 analysis of the growth of ESCC cells when SBI‐581 or cisplatin or vector carrying IRGM plasmid was used.

SBI‐581 is a compound that could specifically inhibit the function of TAOK3 (**Figure** **5**A). It was recently found by lizuka et al.^[^ ^28^ ^]^ Up to date, no one has explored its role in synergizing chemotherapy. After performing the CO‐IP assay, we found the expression of pan‐serine/threonine phosphorylated KMT2C was downregulated significantly when cells were treated with SBI‐581(Figure S6A, Supporting Information). Moreover, the expression of P‐KMT2C (S4588) rather than P‐KMT2C (T4589) was significantly decreased when SBI‐581 was applied, as observed in the results of the Western blot assay (Figure 5B and Figure S6B, Supporting Information). Additionally, we found SBI‐581 could decrease the interaction between KMT2C and ETV5 (Figure 5C). From CCK8 assay, we firstly confirmed the IC_50_ of SBI‐581 concerning to affecting cells growth of ECA109 at 48 h was 2.02 µmol/l, and of KYSE150 was 2.50 µmol/l (Figure S6C,D, Supporting Information). Besides, after SBI‐581 was used, the proliferation of ESCC cells was robustly suppressed from the dynamic growth curve (Figure 5D,E). After the lentivirus vector carrying GFP‐RFP‐LC3 was transfected into ESCC cells, we found the number of autophagy lysosomes decreased when cells were treated with SBI‐581. However, IRGM overexpression could dramatically rescue this effect (Figure 5F,G), which was further validated by the results of TEM (Figure 5H). Meanwhile, the results of the western blot revealed that the expression level of protein LC3 II/I decreased, but the expression level of protein p62 increased when SBI‐581 was used. Besides, IRGM overexpression could rescue this effect significantly (Figure 5I,J). After cells were treated with SBI‐581, the expression level of apoptosis‐associated proteins BAK, BAX, cleaved caspase 3 and cleaved PARP increased, but anti‐apoptosis protein BCL‐2 decreased (Figure 5K,L). All these data imply that SBI‐581 could inhibit autophagy but induce cell apoptosis, which was further verified from the Annexin V‐FITC/PI staining assay (Figure 5M,N). From the CCK8 assay, we found not only could SBI‐581 suppress the survival of ESCC cells but also SBI‐581 could synergize with cisplatin to treat ESCC in vitro. Besides, IRGM overexpression could reverse these effects (Figure 5O,P).

### 2.7 TAOK3 Promotes Tumor Growth and Metastasis in Vivo Together with ETV5 and IRGM, and its Inhibitor Synergizes with Cisplatin In Vivo

**Figure 6:**
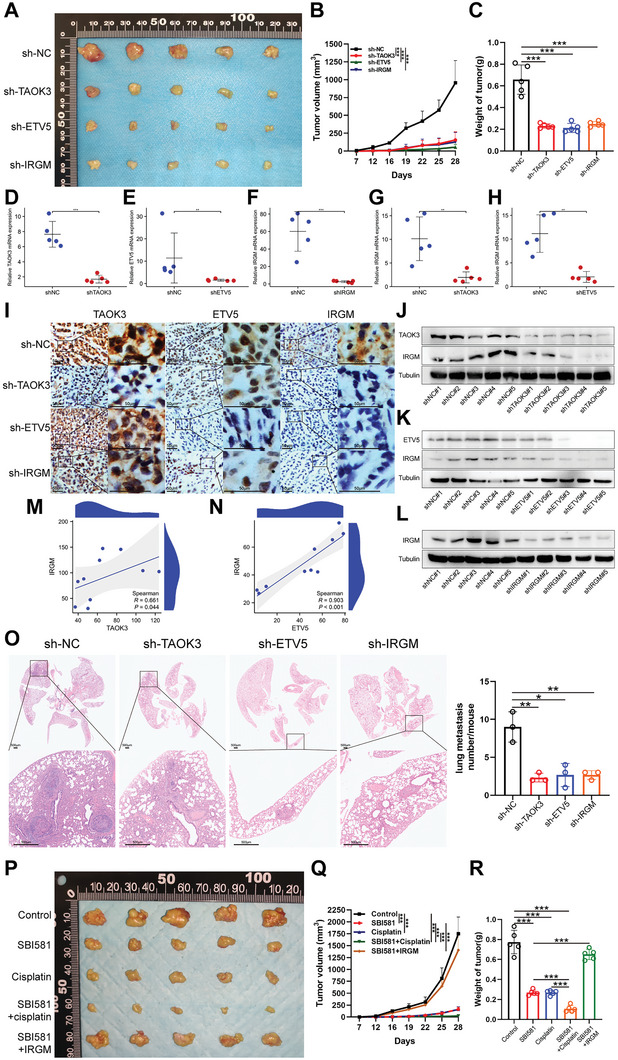
TAOK3, ETV5, and IRGM promote tumor growth and metastasis in vivo, and SBI‐581 synergizes with cisplatin in vivo. A) The image of removed Subcutaneous tumors of mice treated with sh‐TAOK3, sh‐ETV5 or sh‐IRGM. B) The growth curve of Subcutaneous tumors in sh‐TAOK3, sh‐ETV5, and sh‐IRGM groups. C) The weight of Subcutaneous tumors in sh‐TAOK3, sh‐ETV5, and sh‐IRGM groups. D‐F) QPCR analysis of the mRNA expression level of TAOK3, ETV5, and IRGM of removed tumors in sh‐TAOK3, sh‐ETV5, and sh‐IRGM groups respectively. G,H) QPCR analysis of the mRNA expression level of IRGM in sh‐TAOK3 and sh‐ETV5 groups. I) IHC analysis of the expression of protein TAOK3, ETV5, and IRGM of removed tumors in sh‐TAOK3, sh‐ETV5, and sh‐IRGM groups. J—L) Western blot analysis of the expression level of protein TAOK3, ETV5, and IRGM of removed tumors in sh‐TAOK3, sh‐ETV5, and sh‐IRGM groups respectively. M) The correlation analysis of the expression of protein TAOK3 and IRGM in removed tumors. N) Correlation analysis of the expression of protein ETV5 and IRGM in removed tumors. O) HE staining revealed ESCC metastases in the lung tissues. P) The image of removed Subcutaneous tumors of mice in SBI‐581, cisplatin, SBI‐581+cisplatin) and SBI‐581+IRGM groups. Q )The growth curve of Subcutaneous tumors in SBI‐581, cisplatin, SBI‐581+cisplatin, and SBI‐581+IRGM groups. R) The weight of removed tumors in SBI‐581, cisplatin, SBI‐581+cisplatin, and SBI‐581+IRGM groups.

After the tumor growth assay was performed, we found that the tumor growth speed and the weight of the removed tumors were significantly suppressed when TAOK3, ETV5, and IRGM were knocked down respectively (**Figure** **6**A–C). After total RNAs and proteins were isolated from mice tumor tissues, we applied qPCR and western blot assays and found IRGM mRNA and protein were not only downregulated in sh‐IRGM group, but also in sh‐TAOK3 and sh‐ETV5 groups (Figure 6D–L), and the expression of protein IRGM was in positive correlation with TAOK3 or ETV5 (Figure 6M,N), which indicated TAOK3 and ETV5 positively regulated the expression of IRGM. After the tumor metastasis assay was performed, we found TAOK3 or ETV5 or IRGM knockdown could robustly reduce the average number of lung metastasis nodules and the size of metastasis nodules indicated by HE staining (Figure 6O). To evaluate the effects of TAOK3 inhibitor SBI‐581 in vivo, we further performed the tumor growth assay, and found the tumor growth was alleviated when mice were treated with SBI‐581 and cisplatin respectively. SBI‐581 could significantly synergize with cisplatin in vivo. Additionally, we found IRGM overexpression could rescue the effects of SBI‐581 (Figure 6P–R).

### 2.8 TAOK3 and IRGM are Overexpressed in ESCC Tissues and are Associated with Poor Prognosis in ESCC Patients

**Figure 7:**
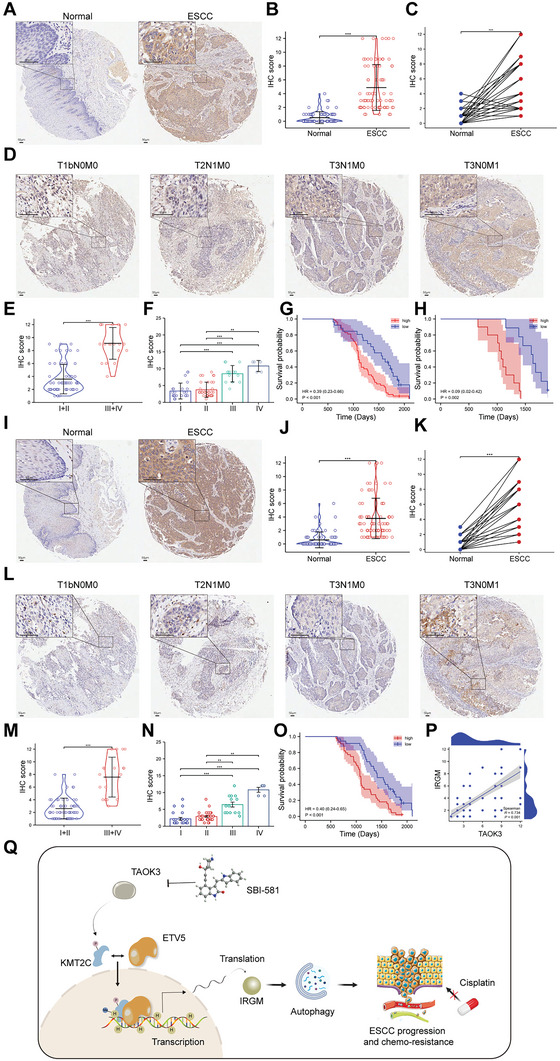
TAOK3 and IRGM are overexpressed in ESCC tissues and are clinically associated with tumor stage and poor prognosis. A,B) IHC analysis of the expression of TAOK3 in ESCC tissues and normal esophageal epithelial tissues. C) IHC analysis of the expression of TAOK3 in ESCC tissues and paired normal esophageal epithelial tissues. D‐F) IHC analysis of the expression of TAOK3 in different TNM stages of ESCC. G) Kaplan‐Meier analysis of the overall survival of ESCC patients with high or low TAOK3 expression. H) Kaplan‐Meier analysis of the overall survival of ESCC patients in metastasis subgroup. I,J) IHC analysis of the expression of IRGM in ESCC tissues and normal esophageal epithelial tissues. K) IHC analysis of the expression of IRGM in ESCC tissues and paired normal esophageal epithelial tissues. L‐N) IHC analysis of the expression of IRGM in different TNM stages of ESCC. O) Kaplan‐Meier analysis of the overall survival of ESCC patients with high or low IRGM expression. P) The correlation analysis of the expression of protein TAOK3 and IRGM in ESCC tissues. Q) The schematic diagram of the underlying mechanism in this study was shown.

After we performed the IHC assay in the ESCC tissue microarray, we found protein TAOK3 was significantly overexpressed in ESCC tissues compared with that in normal esophageal epithelial tissues (**Figure** **7**A–BA). In paired tissues, we also found TAOK3 was overexpressed in ESCC tissues than in paired normal esophageal epithelial tissues (Figure 7C). In this study, we defined metastasis in lymph nodes or other organs as a metastasis group. We found protein TAOK3 was also overexpressed in paired or non‐paired ESCC tissues of the metastasis subgroup (Figure S6E,F, Supporting Information). Furthermore, by analyzing the correlation of clinicopathologic parameters with TAOK3 level in ESCC tissues, we found that TAOK3 protein level was higher in stage III+IV cancers than in stage I+II cancers and positively associated with tumor stage (Figure 7D–F, Supporting Information). Meanwhile, Stratification analysis uncovered that TAOK3 protein expression in ESCC tissues was positively associated with tumor size (*P* = 0.042), metastasis (*P* = 0.002), and TNM stage (*P*<0.001, supplementary table 2). Kaplan–Meier analysis in this study showed that patients whose sample had higher TAOK3 protein levels had shorter overall survival (Figure 7G). We also found patients whose sample had higher TAOK3 protein levels also had shorter overall survival in the metastasis subgroup (Figure 7H). The results of the IHC assay also determined that protein IRGM was overexpressed in ESCC tissues compared with that in paired or non‐paired normal esophageal epithelial tissues (Figure 7I–K). In the metastasis subgroup, the results were the same (Figure S6G‐H). The expression level of IRGM was positively related to tumor stage and metastasis (Figure 7L–N, supplementary table 3). In addition, patients whose sample had higher IRGM protein levels had shorter overall survival (Figure 7O). From correlation analysis, we found the expression level of protein IRGM was positively correlated with protein TAOK3 (Figure 7P).

## 3 Discussion

ESCC patients always have a poor prognosis, mainly due to inapparent symptoms, and prone to metastasis at the early stage.^[^ ^3^, ^4^ ^]^ Up to date, cisplatin is the foremost agent for treating ESCC, but drug resistance has still been an enormous problem. Therefore, figuring out the biological process behind ESCC progression and cisplatin resistance and finding novel therapeutic targets is imperative. Autophagy, a critical homeostatic cellular recycling mechanism, is now emerging as a crucial player in drug resistance.^[^ ^18^ ^]^ Endogenous gene instability and exogenous relative nutrient deficiency are common features of cancers. Facing diverse cellular stresses and maintaining homeostasis simultaneously, 8 hallmarks of cancers were concluded.^[^ ^17^ ^]^ As a cardinal role in resisting cell death, autophagy can inhibit cell apoptosis, help break down cellular organelles and allow the resulting catabolites to be recycled and used for biosynthesis and energy metabolism when certain states of cellular stress occur. As in response to chemotherapy, activating autophagy is a protective mechanism to mediate the acquired resistance phenotype of cancer cells during chemotherapy. Therefore, suppressing autophagy can help re‐sensitize previously resistant cancer cells and synergize with chemotherapy drugs, which was confirmed in many preclinical models.^[^ ^29^, ^30^ ^]^ The increase in autophagy levels in ESCC has been reported in many studies.^[^ ^19^, ^20^, ^21^ ^]^ Apart from PI3K, AKT, and mTOR signaling pathways, MAPK signaling pathway has also been proven to affect autophagy by interacting with several signaling pathways.^[^ ^31^ ^]^

TAO kinases are MAP3Ks belonging to the serine/threonine‐kinase STE20 family.^[^ ^7^, ^8^ ^]^ TAO kinases act as crucial mediators in response to various stimuli, including DNA damage and nutrition deficiency. They are reported to augment chemotherapy resistance in many cancers,^[^ ^15^, ^16^ ^]^ but the underlying mechanism is still unclear. Apart from regulating various signaling pathways, TAO kinases are reported to interact with other cytosolic proteins and regulate the DNA damage responses, cytoskeleton stability, apoptosis, and other physiological and pathophysiological responses.^[^ ^8^ ^]^ In our study, we first found TAOK3, rather than TAOK1 and TAOK2 was significantly overexpressed in ESCC cell lines and tissues. Besides, only TAOK3 could augment autophagy in ESCC. Functionally, TAOK3 could promote proliferation, migration, invasion, and cisplatin resistance, but suppress cell apoptosis in ESCC cells. All these results indicate that TAOK3 promotes ESCC progression and cisplatin resistance by inducing autophagy. Mechanistically, TAOK3 can phosphorylate a histone methyltransferase KMT2C at S4588 and strengthens the interaction between KMT2C and the transcription factor ETV5. Consequently, the nuclear translocation of KMT2C is increased, and the transcription of IRGM is upregulated with the help of H3K4Me3. The increase in autophagy is mediated by IRGM upregulation, similar to other studies.^[^ ^32^, ^33^ ^]^ Clinically, the expression level of TAOK3 was positively correlated to the tumor stage but negatively correlated to overall survival. These results demonstrated that TAOK3 could be viewed as an oncogene and prognostic biomarker in ESCC, similar to other cancers.^[^ ^16^, ^34^, ^35^ ^]^ The correlation analysis showed the expression level of protein IRGM was positively correlated with protein TAOK3, which indicated that the expression of IRGM was regulated by TAOK3 in ESCC tissues.

Considering that TAOK3 promotes ESCC progression in this study dependent on its kinase activity, we look forward to seeking out specific inhibitor for TAOK3 in order to find a way to ameliorate their effects on tumor progression. Recently, after scanning from 5600 compounds, Lizuka et al. found a specific inhibitor named SBI‐581 (IC_50_ = 42nm) for TAOK3.^[^ ^28^ ^]^ However, no one ever explored its tumor inhibition effect, especially synergistic action with chemotherapy. Our study found that SBI‐581 could evidently migrate the phosphorylation from TAOK3 to KMT2C, and the binding between KMT2C and ETV5 was released robustly. After SBI‐581 was used, the autophagy level in ESCC cells was attenuated, and IRGM overexpression could effectively rescue this effect. From in vitro and in vivo assays, the synergistic effect of SBI‐581 with cisplatin was confirmed. All these results indicate that SBI‐581 can significantly inhibit TAOK3's effect on ESCC and commendably synergize with cisplatin concerning to treating ESCC. Thus, SBI‐581 has an excellent possibility to be used to treat ESCC clinically in the future. There are still some limits in our study. Though through MS and generating specific antibodies, we identified that TAOK3 phosphorylates KMT2C at S4588, it is challenging for us to perfect this work by performing site mutation or enzymatic assays due to the large molecular weight of KMT2C. Besides, the detailed mechanism behind autophagy stimulation by IRGM in ESCC is worth further exploration.

## 4 Conclusion

In conclusion, we identified a potential role of TAOK3 in the progression and chemoresistance of ESCC for the first time. The overexpression of TAOK3 in ESCC could upregulate the expression of autophagy‐relevant protein IRGM and further augment autophagy in ESCC. Mechanistically, TAOK3 phosphorylates KMT2C at S4588 and promotes the interaction between KMT2C and ETV5. The nuclear translocation of KMT2C was increased, and the transcription of IRGM was upregulated with the help of KMT2C and ETV5. TAOK3 could serve as an oncogene and a vital prognostic biomarker in ESCC. The inhibitor SBI‐581 could serve as a novel treatment strategy together with cisplatin.

## 5 Experimental Section

### Cell Culture and Reagents

ESCC cell lines, including ECA9706, KYSE30, TE1, KYSE510, KYSE410, ECA109, and KYSE150, were cultured in RPMI 1640 medium (GibCo) supplemented with 10% FBS (GibCo) in an incubator containing 5% CO2 at 37 °C. All cell lines were purchased from the Institute of Biochemistry and Cell Biology of the CAS (Shanghai, China).

### Bioinformatics Analysis

The Cancer Genome Atlas (TCGA) is a large‐scale cancer genomics program, and it has molecularly characterized 33 primary cancer types comprising esophageal carcinoma. This study investigated the expression of TAO kinases in esophageal carcinoma from the TCGA.

### Patients Tissue Specimens

A total of 79 paired ESCC tissue specimens from both tumor and corresponding non‐tumorous tissues were collected from untreated patients who underwent surgical treatment for ESCC at Shanghai East Hospital, affiliated with Tongji University. Written informed consent was obtained from each patient, and the Ethics Committee of Shanghai East Hospital approved the investigation (Approval number: 2019061). All samples were cut into two parts. One part was embedded in paraffin and processed for immunohistochemical analysis, while the other part was frozen immediately in liquid nitrogen and stored at −80 °C for further studies.

### Immunohistochemical (IHC) Analysis

Tissue microarray (TMA) sections were made from tumor and nontumor tissues of enrolled ESCC patients. After the sections were placed in xylene and graded alcohols for deparaffinization and hydration, heat‐induced antigen retrieval in EDTA (PH 8.0) buffer for 15 min was performed in a microwave oven. Blocking with 10% goat serum for reducing nonspecific staining was carried out. The primary antibodies were anti‐TAOK3 (#67451‐1‐lg, Proteintech) and anti‐IRGM (#AP11128b, Abcepta). The expression level of TAOK3 and IRGM were evaluated and obtained under a ZEISS microscope.

### Cell Transfection

For transient gene knockdown transfection, cells were plated in 6‐well plates and transfected with specific siRNA for TAOK3, ETV5, or IRGM (GenePharma, Shanghai, China) following the manufacturer's instruction. For transient gene overexpression transfection, the plasmid pcDNA3.1‐FLAG‐TAOK3, pcDNA3.1‐FLAG‐ETV5, pcDNA3.1‐IRGM, and the parental control vector were purchased from Genechem (Shanghai, China). Real‐time quantitative polymerase chain reaction (RT‐qPCR) and western blotting were applied to detect both knockdown and overexpression efficiency at 48 h after transfection. For stable transfection, lentivirus vectors carrying shTAOK3, shETV5, shIRGM, GFP‐RFP‐LC3 (Genechem, Shanghai, China), or IRGM was packaged and transfected into ESCC cells, and transfected cells were selected with puromycin (Yeasen).

### Monodansyl Cadaverine (MDC) Staining

To primarily evaluate autophagy, fluorescent probe MDC was applied, a selective marker for acidic vesicular organelles including autophagic vacuoles. Briefly, the transfected ECA109 cells grown on coverslips were exposed to MDC (Beyotime) for 40 min at 37 °C in the dark for staining. After washing with Assay Buffer 3 times, cells were observed by a fluorescence microscope (ZEISS).

### Colony Formation Assay

One thousand transfected cells were seeded into 6‐well plates and maintained at 37 °C in a 5% CO2 incubator for 2 weeks. 4% paraformaldehyde was used to fix the colonies. 0.5% crystal violet was used to stain the colonies. The number of colonies were counted under a microscope. The independent experiments were performed in triplicate.

### CCK8 Assay

We applied CCK8 assay to evaluate the proliferation capacity of ESCC cells and the sensitivity of ESCC cells to cisplatin. In brief, 1 × 10^3^ cells were plated in each well of 96‐well plates. To evaluate the proliferation capacity, at 0, 24, 48, 72, and 96 h, 10 µl CCK8 reagent was added to each well and incubated with ESCC cells for 2 h. The absorbance of ESCC cells in each well was measured by a microplate reader. To determine cisplatin sensitivity, the absorbance was measured after cells were treated by cisplatin (MCE) for 48 h.

### Edu Staining Assay

Following the manufacturer's instructions, cell proliferation by Edu reagent (Beyotime) was monitored. Nuclear detection was determined by DAPI staining, and the cells were observed by a fluorescence microscope (ZEISS).

### Transwell Assay

Transwell assays were performed to evaluate cell migration and invasion abilities. To explore the migration ability, 200 µl RPMI 1640 without serum was used to resuspend ESCC cells and added them in the upper chamber of the transwell device, with 5 × 10^4^ cells/well. Subsequently, 600 µl complete medium was added into the lower chamber as the chemical attractants. After incubation for 48 h at 37 °C, cells on the lower surface of the non‐coated membrane were fixed by 4% paraformaldehyde and then stained by Giemsa. Images from five representative fields of each membrane were taken by using a light microscope (100×). The number of migratory cells was counted and the relative migration rate was calculated. Invasion assay was similar to migration assay, only with the difference that 100 µl of 200µg ml^−1^ diluted Matrigel matrix (Corning, 356234) was carefully added to the center of each transwell insert before cells were added and incubated at 37 °C for 2 h to form a gel layer.

### Wound Healing Assay

The ECA109 and KYSE150 cells were seeded onto a 6‐well plate, and when the cell density reached 80%, a sterile 200 µl pipette tip was used to make a straight scratch line on the confluent cell monolayer. The medium was replaced with the 1% FBS‐contained medium. At 0, 24, and 48 h after scratch, cell migration was imaged by a light microscope (100×). Image J was used to calculate the closure.

### Autophagic Vesicle Imaging by Transmission Electron Microscopy (TEM)

The harvested cells were fixed with 4% glutaraldehyde and then fixed with 1% OsO_4_ in 0.1 M cacodylate buffer containing 0.1% CaCl_2_ at 4 °C for 2 h. We then stained the cells with 1% uranyl acetate, dehydrated the samples in increasing concentrations of ethanol, and embedded the samples in Araldite. After the thin sections were stained with uranyl acetate and lead citrate, the authors analyzed them with an electron microscope (Tecnai G2, FEI) at 11500 magnifications.

### Annexin V‐FITC/PI Staining Assay

After cells were harvested and washed with cold PBS. The Annexin V‐fluorescein isothiocyanate (FITC) and Propidium Iodide (PI) apoptosis detection kit (Dojindo) was applied to quantitatively estimate the level of phosphatidylserine on cell surface according to the manufacturer's instruction. A flow cytometer (Beckman) was used to analyze the apoptotic cells. A minimum of 10 000 events was collected for each sample. The percentage of apoptotic cells to total cells was shown as the results.

### Real‐Time qPCR Assay and RNA Sequencing

Total RNAs were isolated using Trizol reagent (Invitrogen CA, USA). RNA concentrations were measured by Nanodrop2000 spectrophotometer, and complementary DNA (cDNA) was synthesized according to the instruction of the PrimeScript RT reagent kit (Takara, Japan). Real‐time qPCR (TaqMan) for TAO kinases, ETV5, and IRGM was performed following the instruction of Takara Bio, and β‐actin served as an internal reference. qPCR cycles were as follows: denaturation for 5 min at 95 °C, 30 cycles of 5 s at 95 °C, and extension at 65 °C for 34 s. The 2^−△△CT^ method was used to calculate target values by standardizing to the internal reference (average of controls). The relevant primers were summarized in supplementary Table 1. Library preparation for RNA sequencing was conducted. Generally, 1 µg high‐quality RNA was used, and sequencing was carried out by HiSeq2500 (Illumina Inc., San Diego, CA) at Genechem Biotechnology (Shanghai) Co., Ltd.

### Western Blotting Analysis

RIPA buffer supplemented with 1% PMSF and protease inhibitor cocktail was used to extract protein from both cell lines and tissues. The protein concentrations were measured using BCA assay (Beyotime). Different Samples were separated on 6%–15% SDS‐PAGE gels, followed by protein transfer onto the PVDF membrane (Millipore, USA). After treatment with 5% nonfat milk, the membranes were probed with diluted primary antibodies against β‐Actin (#8457, CST), GAPDH (#5174, CST), β‐Tubulin (#2146, CST), TAOK3 (#ab70297, Abcam), KMT2C (#28437‐1‐AP, Proteintech), ETV5 (#ab102010, Abcam), IRGM (#AP11128b, Abcepta), H3K4me3 (#9727, CST), LC3B (#ab192890, Abcam), P62 (#ab109012, Abcam), FLAG tag (#66008‐4‐lg, Proteintech), Cleaved PARP (#5625, CST), Caspase‐3 (#A2156, Abclonal), BCL‐2 (#A19693, Abclonal), BAX (#41162, CST), BAK (#A10754, Abclonal), Lamin B (#A11459, Abclonal), P‐KMT2C‐S4588 (#E25915, generated from Abclonal) and P‐KMT2C‐T4589 (#E25804, generated from Abclonal) overnight at 4 °C. After the membranes were washed three times with TBST for 10min each, the membranes were incubated by corresponding HRP‐labeled secondary antibody for 2h at room temperature. Signals were examined using an ECL detection system (Tanon).

### Immunofluorescence (IF)

A 4% paraformaldehyde was used to fix cells for 15 min at room temperature. Fixed cells were permeabilized with 0.5% Triton‐X100 for 20 min. 5% BSA was used to block cells for an hour, and then the cells were incubated with relevant primary antibodies against KMT2C (#28437‐1‐AP, Proteintech) and ETV5 (#sc‐100941, Santa cruz) at 4 °C overnight. Fluorescein isothiocyanate‐conjugated secondary antibody was used to incubate cells for 60 min at room temperature. After washing with PBS for three times, the cells were counterstained with DAPI for 15 min in the dark. Images were captured by fluorescence microscope (ZEISS) or confocal laser scanning microscope (Olympus). Fluorescence density was calculated with ImageJ software.

### Co‐Immunoprecipitation (CO‐IP) Assay

After the protein was extracted, the lysate was precleared using a mixture of protein A/G plus‐agarose (sc‐2003, Santa Cruz) and the specific primary antibody or control immunoglobulin IgG (#sc‐2025, Santa Cruz and #AC005, Abclonal) on a rotating device overnight at 4 °C. Immunoprecipitates were washed with ice‐cold lysis buffer four times, resolved by SDS PAGE, and analyzed by western blot.

### Silver Staining and Mass Spectrometry Analysis

Similar to the CO‐IP assay, cellular protein extracts from ECA109 cells were incubated with anti‐TAOK3 and anti‐ETV5, respectively, followed by protein A/G agarose beads. Recovered proteins associated with TAOK3, ETV5 or IgG were resolved by SDS PAGE. Silver staining was performed following the manufacturer's suggestions (Beyotime). The bands specifically bound were excised to TAOK3 or ETV5 and finished proteomics screening by mass spectrometry analysis on a MALDI‐TOF‐MS instrument (Bruker Daltonics).

### Chromatin Immunoprecipitation (CHIP) Assay

About 1×10^7^ ECA109 cells were cross‐linked with 10% formaldehyde for 10 min and quenched by glycine. After washing with PBS 3 times, the cells were harvested in CHIP lysis buffer (50 mM Tris‐HCL, PH 7.6, 1 mM CaCl_2_, 0.2% Triton X‐100). MNase was used to digest samples, and sonication followed. Sonicated DNAs were incubated with protein G beads and anti‐KMT2C or anti‐H3K4Me3 or anti‐ETV5 or IgG at 4 °C overnight. CHIP wash buffer was used to wash incubated beads 5 times, and DNA was eluted by CHIP elution buffer (0.1 M NaHCO_3_, 1% SDS, 20µg ml^−1^ proteinase K). After de‐crosslinking, a DNA purification kit (TIANGEN DP214‐03) was used to extract DNA. The amount of bound DNA was measured by qPCR. The relative enrichment was normalized to input. The primers covering KMT2C, H3K4Me3, and ETV5 binding sites of the IRGM gene promoter region were summarized in supplementary Table 1.

### Animal Studies

Under the approval of the animal care and use committee of Tongji University (Approval number: TJBB05222101), it was performed in vivo studies, including tumor growth assay and tumor metastasis assay. For in vivo tumor growth assay, stable transfected ECA109 cells (5 × 10^6^) were harvested and subcutaneously injected into the flanks of well‐euthanized female BALB/c nude mice (6 weeks old). Tumors’ size was measured by a caliper every 3 days. The tumor volume was calculated using the following formula: tumor volume (mm^3^) = length × width × width /2. At 28 days, according to the AVMA Guidelines for the Euthanasia of Animals, we performed an intraperitoneal injection of a three‐fold dose of barbiturates to euthanize all the mice. We then removed the tumor and weighed the weight. Subsequently, the tumor was cut into two pieces average. One piece was fixed with 10% formaldehyde and waited for IHC staining. The other piece was prepared for western blotting analysis. To evaluate the effects of inhibitor, SBI‐581 and cisplatin on ESCC in vivo, we inject SBI‐581 (10mg/kg/1 day)^[^ ^28^ ^]^ or cisplatin (3mg/kg/2 days)^[^ ^36^ ^]^ for 14 days after the tumor forms for 12 days. For in vivo tumor metastasis assay, stable transfected ECA109 cells (5 × 10^6^) were injected from the tail vein of the nude mice. Six weeks after injection, the lungs of euthanized mice were removed, and lung metastasis was detected. After lung removal, all lungs were also fixed with 10% formaldehyde and prepared for HE staining.

### Statistical Analysis

All assays were performed in biological triplicate, and SPSS 19.0 (IBM, Armonk, NY, USA) was used to perform statistical analysis. Group differences were estimated by the Student's t‐test, Chi‐square test, or Fisher's exact test. Spearman or Pearson's method was performed to analyze the correlation. Kaplan Mier methods and log‐rank test were used to analyze survival data. *P* <0.05 was viewed as statistical significance.

## Conflict of Interest

The authors declare no conflict of interest.

## Author Contributions

S.M.C. and L.Z.X. contributed equally to this work. X.M.D. and C.T. designed this study and revised the article. S.M.C., L.Z.X., W.X.Y., and Z.M.R. performed the experiments. C.Y., Z.Z.Y., and Z.L. collected clinical specimens and patients’ information. Z.Z.H. and F.K. carried out clinicopathological analysis. F.A.Q. and L.Z.Y. performed bioinformatics analysis. S.M.C., L.Z.X., and S.J.N. analyzed the data. S.M.C. and C.T. wrote the paper. All authors read and approved the final manuscript.

## Supporting information

Supporting Information

## Data Availability

The data that support the findings of this study are available from the corresponding author upon reasonable request.
