# Supplementary material for: TAOK3 Facilitates Esophageal Squamous Cell Carcinoma Progression and Cisplatin Resistance Through Augmenting Autophagy Mediated by IRGM
Source: Adv Sci (Weinh). 2023 Sep 13;10(29):2300864. doi: 10.1002/advs.202300864 (PMC10582451; doi:10.1002/advs.202300864)
Supplement: Supplementary file 1 — Supporting Information [file ADVS-10-2300864-s001.pdf]

ADVANCED SCIENCE
Open Access

Supporting Information

for *Adv. Sci.*, DOI 10.1002/advs.202300864

TAOK3 Facilitates Esophageal Squamous Cell Carcinoma Progression and Cisplatin Resistance Through Augmenting Autophagy Mediated by IRGM

Mingchuang Sun, Zhaoxing Li, Xiaoyuan Wang, Meirong Zhao, Yuan Chu, Zehua Zhang, Kang Fang, Ziying Zhao, Anqi Feng, Zhuyun Leng, Jianing Shi, Li Zhang, Tao Chen and Meidong Xu**

Supporting information

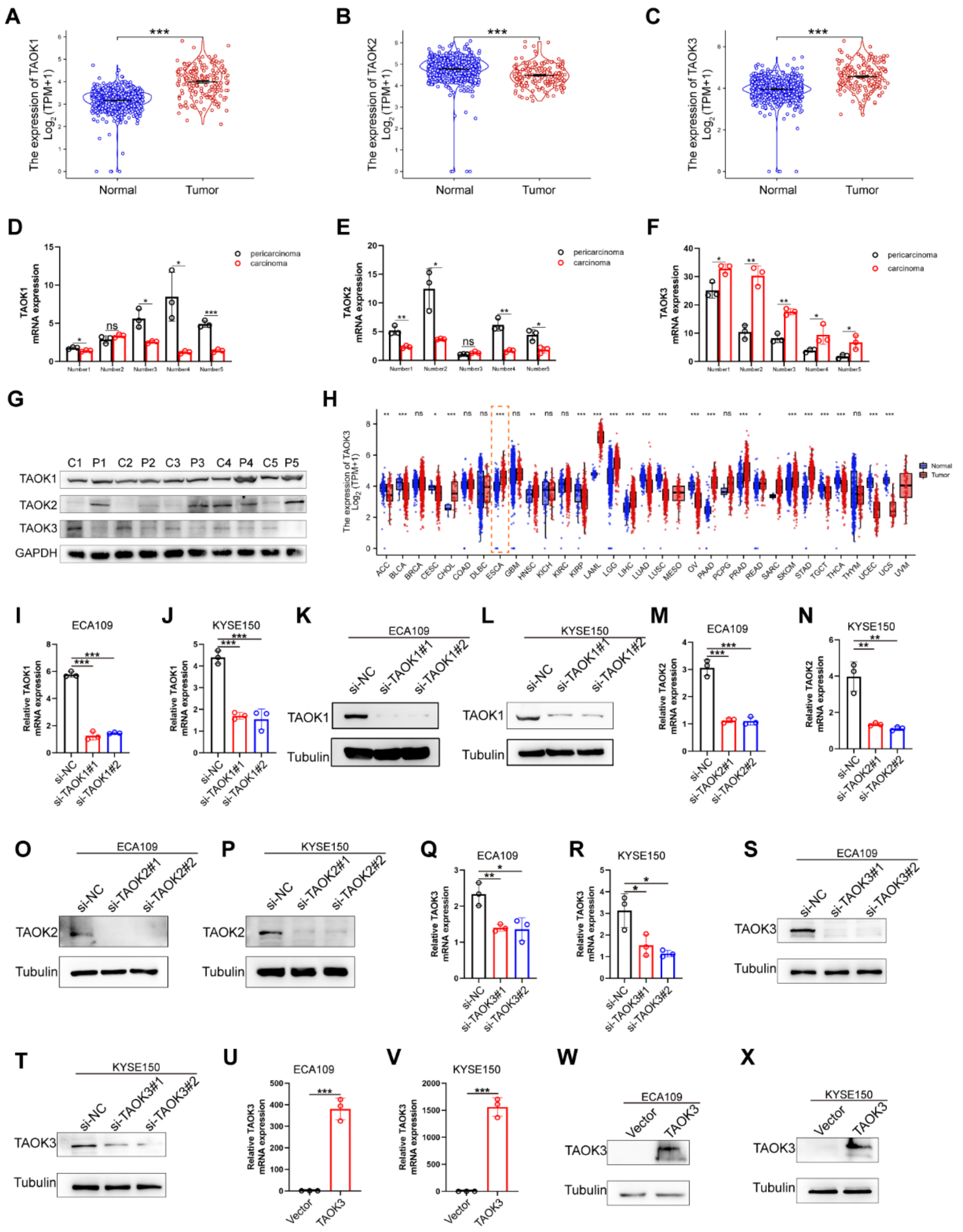

Fig. S1 TAOK3 is overexpressed in ESCC tissues from TCGA analysis and our samples, and the knockdown and overexpression efficiency of TAO kinases is high. A-C TCGA database analysis of the mRNA expression level of TAOK1, TAOK2, and TAOK3. D-F QPCR analysis of the

mRNA expression level of TAO kinases in 5 pairs of ESCC tissues. G Western blot analysis of the protein expression level of TAO kinases in 5 pairs of ESCC tissues. H TCGA analysis of pan-cancer mRNA expression of TAOK3. I-T QPCR and western blot analysis of the knockdown efficiency of siRNAs targeting TAOK1, TAOK2, and TAOK3. U-X QPCR and western blot analysis of the overexpression efficiency of pcDNA3.1-FLAG-TAOK3 transfection.

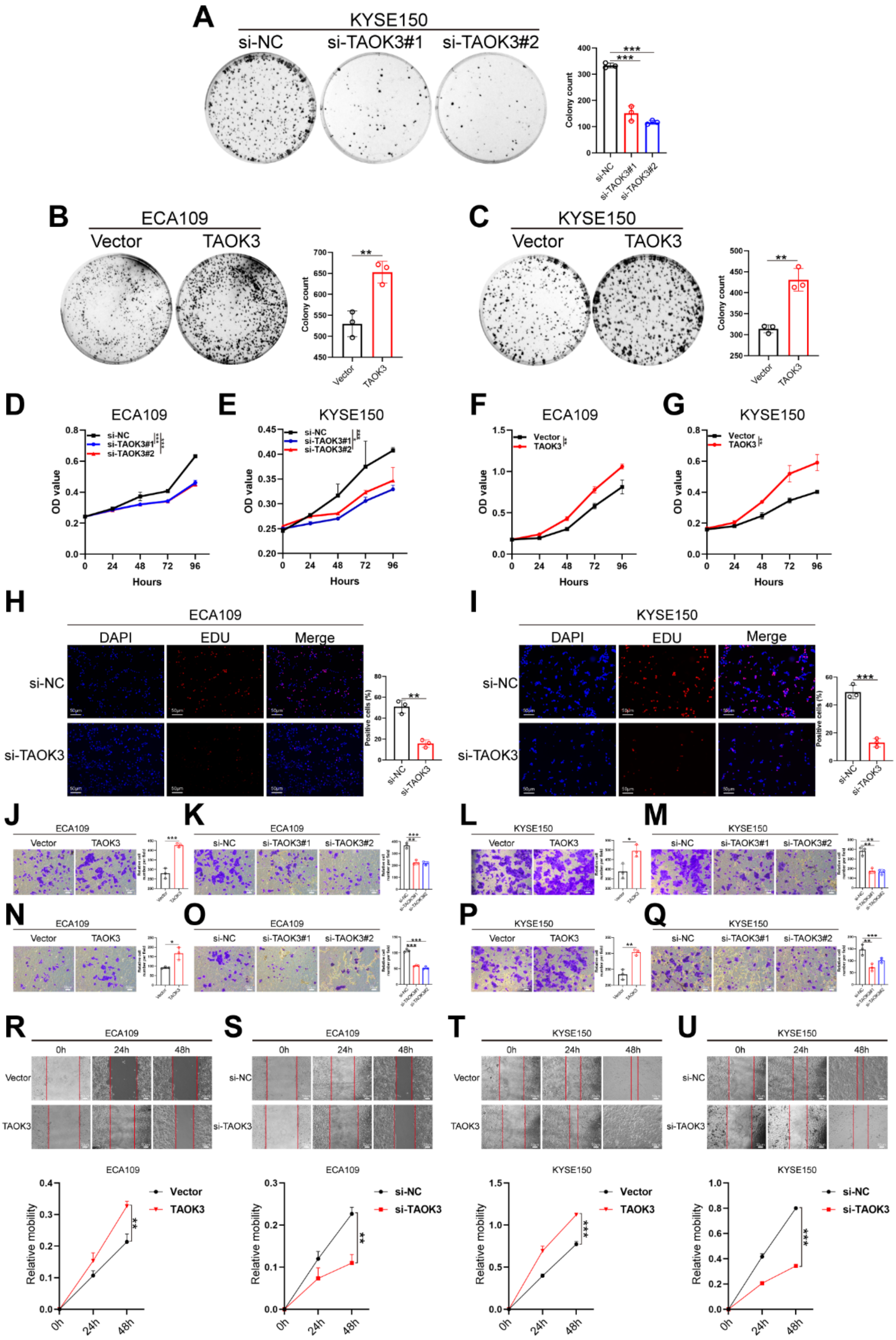
A
KYSE150
si-NC
si-TAOK3#1
si-TAOK3#2
Colony count
B
ECA109
Vector
TAOK3
Colony count
C
KYSE150
Vector
TAOK3
Colony count
D
ECA109
E
KYSE150
F
ECA109
G
KYSE150
OD value
Hours
H
ECA109
DAPI
EDU
Merge
si-NC
si-TAOK3
Positive cells (%)
I
KYSE150
J
ECA109
K
ECA109
L
KYSE150
M
KYSE150
N
ECA109
O
ECA109
P
KYSE150
Q
KYSE150
R
ECA109
S
ECA109
T
KYSE150
U
KYSE150
0h
24h
48h
Relative mobility

Fig. S2 TAOK3 promotes proliferation, migration and invasion in vitro. A-C Colony formation analysis of the proliferation of ESCC cells when TAOK3 was overexpressed or knocked down. D-G CCK8 analysis of the viability of ESCC cells when TAOK3 was overexpressed or knocked down. H-I Edu analysis of the proliferation of ESCC cells when TAOK3 was overexpressed. J-M Migration assay showed the alteration when TAOK3 was overexpressed or knocked down. N-Q Invasion assay showed the alteration when TAOK3 was overexpressed or knocked down. R-U Wound healing analysis of the migration and invasion of ESCC cells when TAOK3 was overexpressed or knocked down.

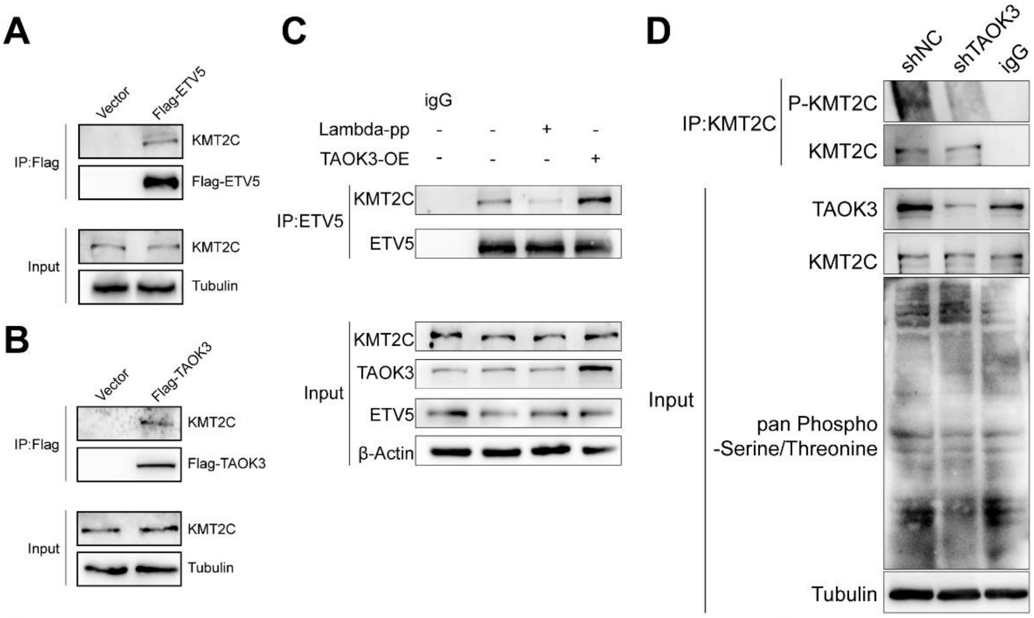

E

Sequence	Modifications	Site	Master Protein Accessions	Theo. MH+ [Da]	Score
LYWSTRYANRR	1xPhospho [Y/S/T]	S4588;T4589	Q8NEZ4	1565.737	1.24
TLVLSDKHSPQKK	1xPhospho [S5(77.9)]	S2807	Q8NEZ4	1560.815	1.12
KDIDDNSNGTYEK	1xPhospho [S/T/Y]	S177;T180	Q8NEZ4	1578.632	0.87
KFSPGRPRSK	1xPhospho [S3(90)]	S836	Q8NEZ4	1239.636	0.84
SLGLSR	1xPhospho [S5(80.9)]	S1985	Q8NEZ4	712.3389	0.71
KFSPGRPR	1xPhospho [S3(100)]	S836	Q8NEZ4	1024.509	0.68
QSGEDSK	1xPhospho [S2(99.7)]	S397	Q8NEZ4	830.2928	0.55
SEPKMSAHVK	1xPhospho [S1(94.6)]	S4711	Q8NEZ4	1193.538	0.47
KMKTEWK	1xPhospho [T4(100)]	S4767	Q8NEZ4	1030.479	0.46
DLTLLNKDSR	1xPhospho [T/S]	T4218;S4224	Q8NEZ4	1254.609	0.45

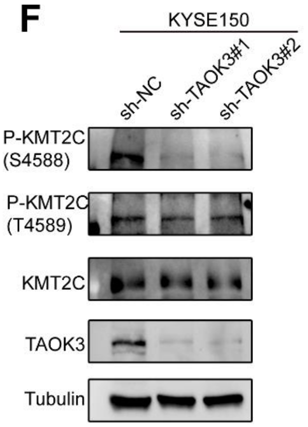

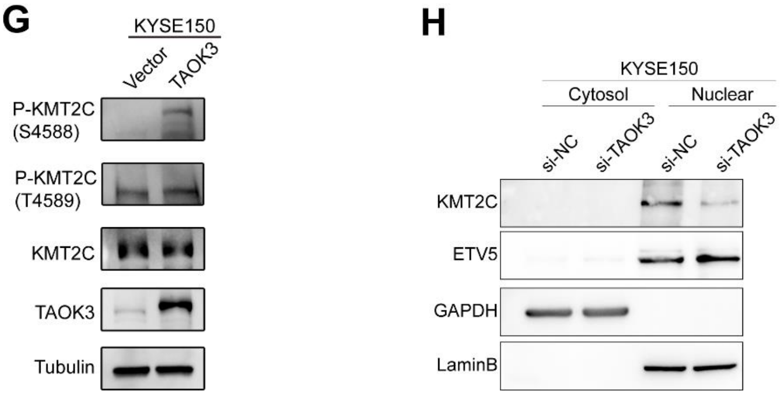

Fig. S3 TAOK3 phosphorylates KMT2C at S4588. A-B Exogenous CO-IP analysis of the interaction between TAOK3, KMT2C, and ETV5 in ECA109 cells. C CO-IP analysis of the interaction between KMT2C and ETV5 in ECA109 cells after L-PP was used, or TAOK3 was overexpressed. D CO-IP analysis of the expression of the pan-serine/threonine phosphorylated KMT2C in ECA109 cells when TAOK3 was knocked down. E MS analysis of the potential phospho-sites of TAOK3 to KMT2C. F Western blot analysis of the expression of P-KMT2C (S4588) and P-KMT2C (T4589) when TAOK3 was knocked down in KYSE150 cells. G Western blot analysis of the expression of P-KMT2C (S4588) and P-KMT2C (T4589) when TAOK3 was overexpressed in KYSE150 cells. H Western blot analysis of nuclear and cytosol fractionated proteins in KYSE150 cells.

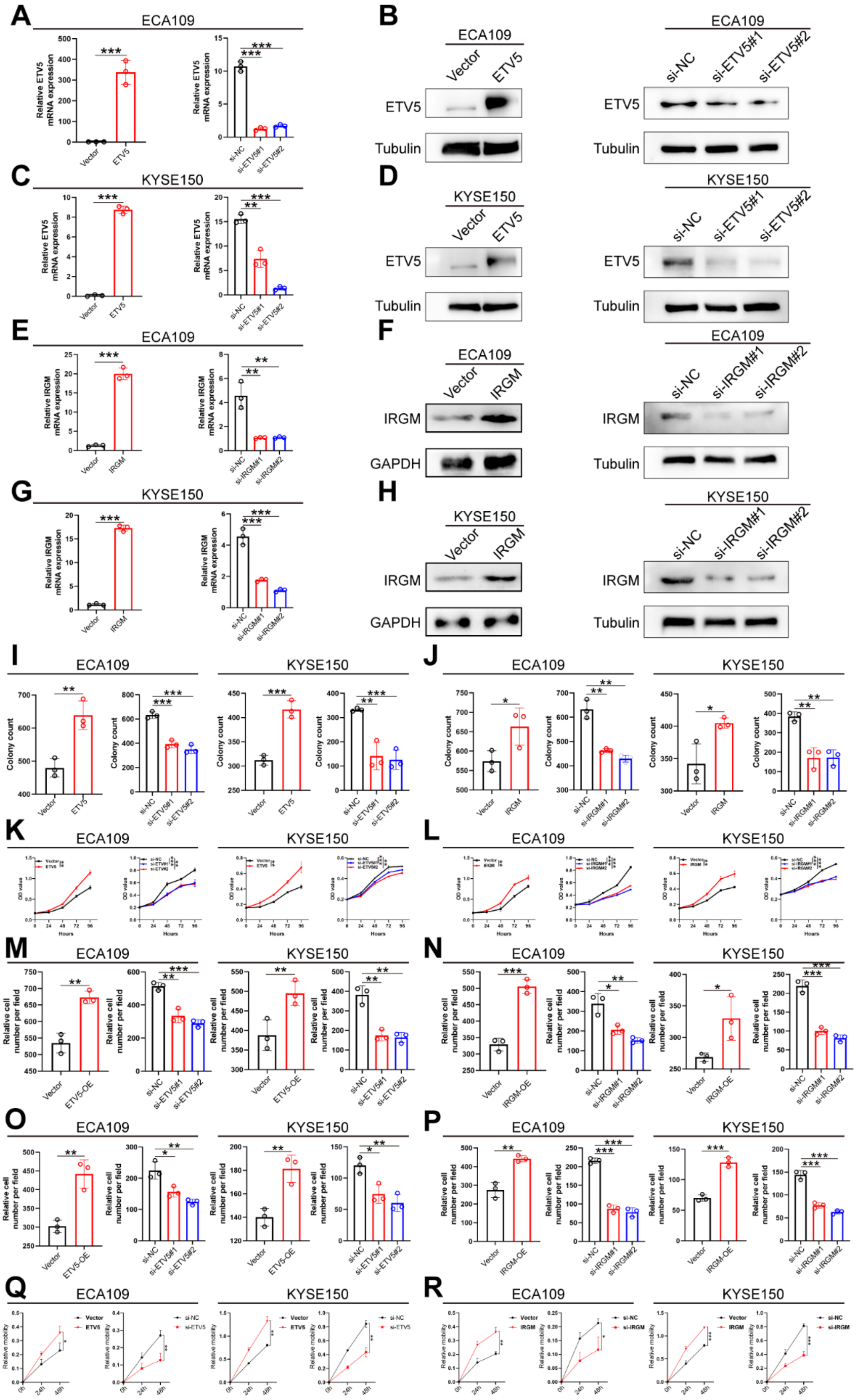
A
ECA109
Relative ETV5 mRNA expression
Vector
ETV5
si-NC
si-ETV5#1
si-ETV5#2
B
ECA109
Vector
ETV5
Tubulin
si-NC
si-ETV5#1
si-ETV5#2
C
KYSE150
D
KYSE150
E
ECA109
Relative IRGM mRNA expression
IRGM
si-IRGM#1
si-IRGM#2
F
GAPDH
G
KYSE150
H
I
Colony count
J
K
OD value
Hours
L
M
Relative cell number per field
ETV5-OE
N
IRGM-OE
O
P
Q
Relative mobility
R
si-IRGM

Fig. S4 ETV5 and IRGM promote proliferation, migration and invasion in vitro. A-H QPCR and western blot analysis of the knockdown and overexpression efficiency of ETV5 and IRGM. I-J Colony formation analysis of the proliferation of ESCC cells when ETV5 or IRGM was overexpressed or knocked down. K-L CCK8 analysis of the viability of ESCC cells when ETV5 or IRGM was overexpressed or knocked down. M-N Migration assay showed the alteration when ETV5 or IRGM was overexpressed or knocked down. O-P Invasion assay showed the alteration when ETV5 or IRGM was overexpressed or knocked down. Q-R Wound healing analysis of the migration and invasion of ESCC cells when ETV5 or IRGM was overexpressed or knocked down.

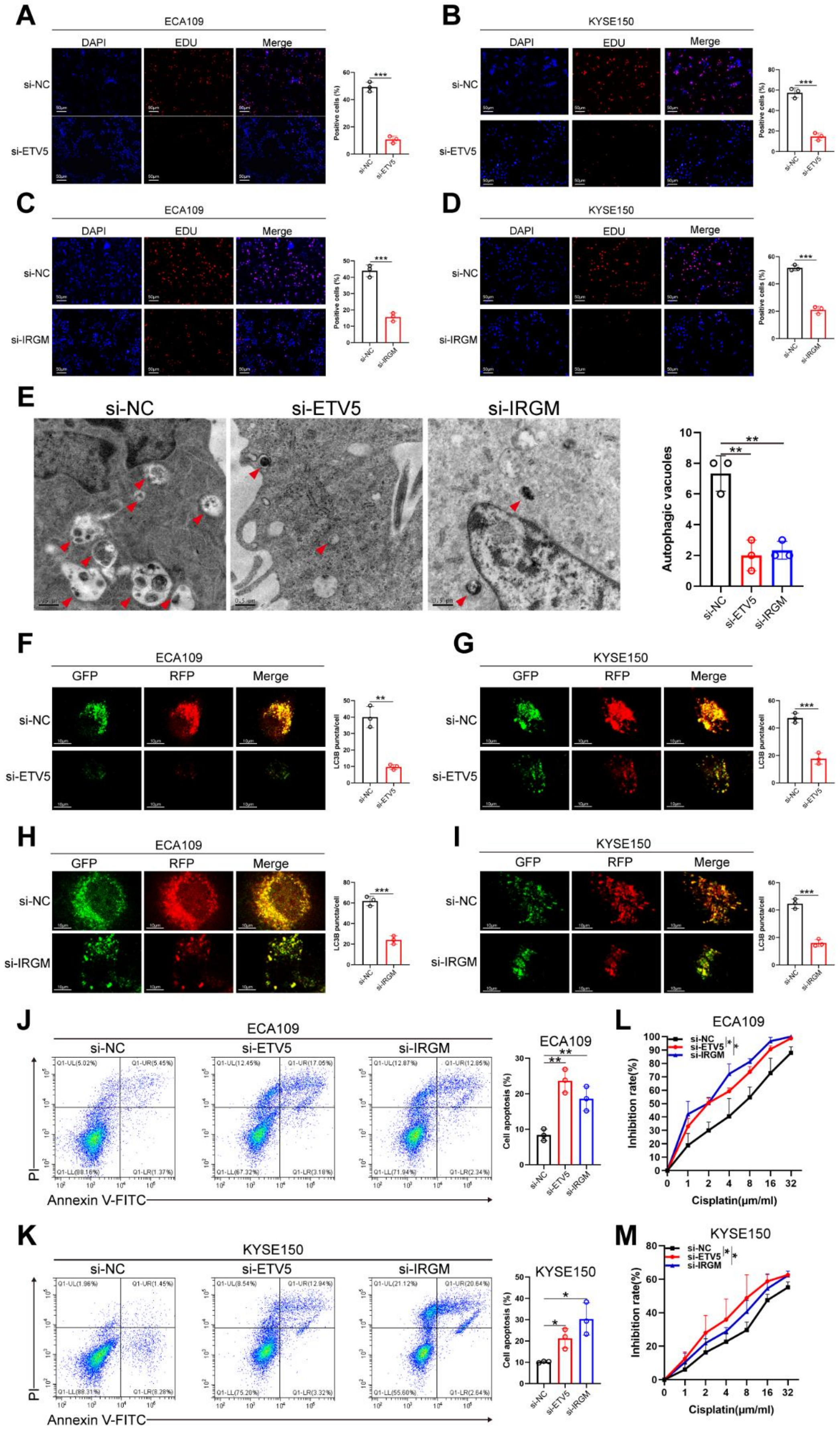
A
ECA109
DAPI
EDU
Merge
si-NC
si-ETV5
B
KYSE150
C
si-IRGM
D
E
si-NC
si-ETV5
si-IRGM
Autophagic vacuoles
F
GFP
RFP
LC3B puncta/cell
G
H
I
J
Annexin V-FITC
PI
Cell apoptosis (%)
K
L
Inhibition rate(%)
Cisplatin(μm/ml)
M

Fig. S5 ETV5 and IRGM promote autophagy and cisplatin resistance but inhibit cell apoptosis in vitro. A-D Edu analysis of the proliferation of ESCC cells when ETV5 or IRGM was knocked down. E TEM analysis of Autophagic vesicle formation when ETV5 or IRGM was knocked down in ECA109 cells. F-I Confocal microscopy analysis of autophagy-lysosome formation when ETV5 or IRGM was knocked down after lentivirus vector carrying GFP-RFP-LC3 was transfected. J-K Annexin V-FITC/PI staining analysis of cell apoptosis level when ETV5 or IRGM was knocked down in ESCC cells. L-M CCK8 analysis of the inhibition rate of ESCC cells growth when ETV5 or IRGM was knocked down.

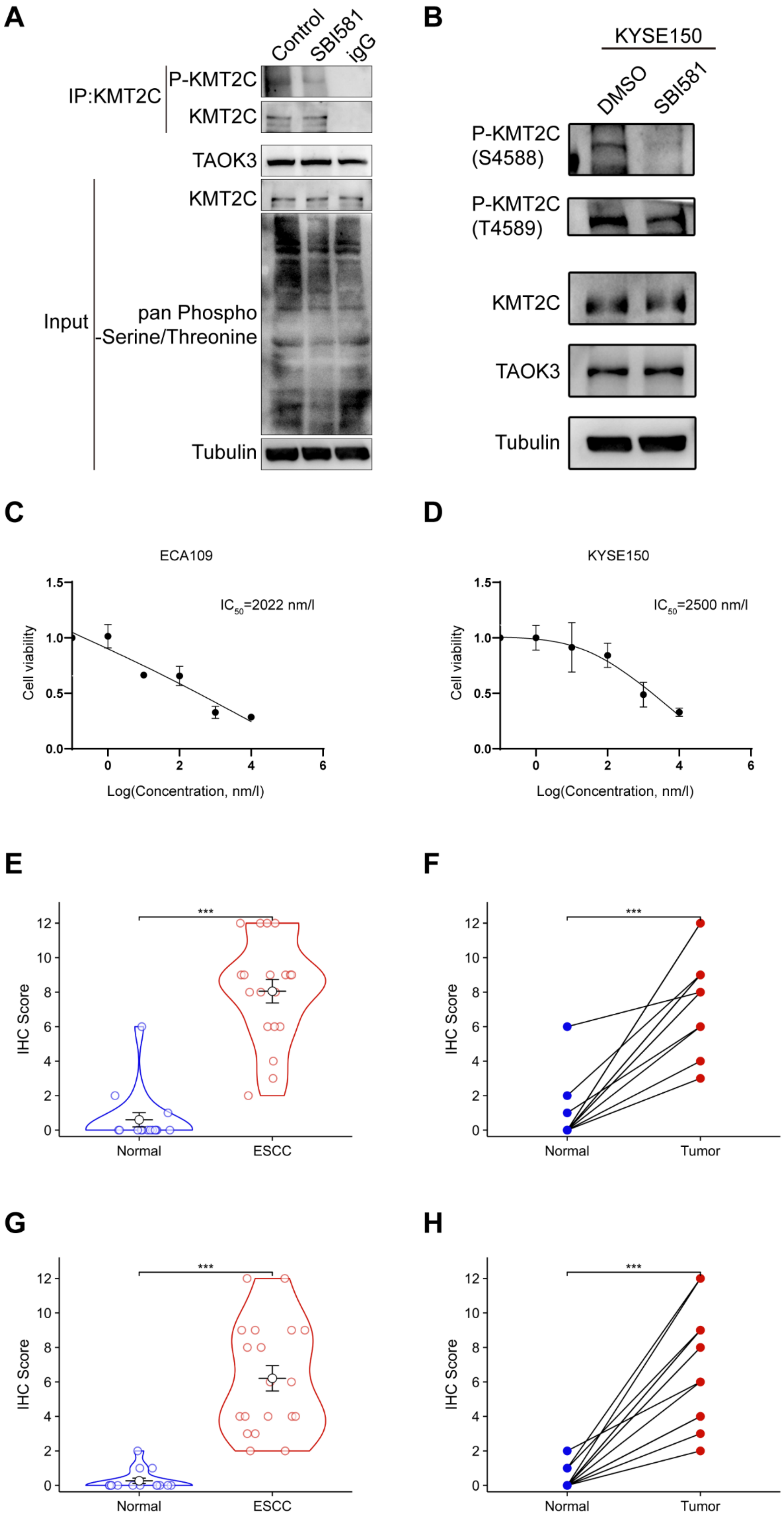

A
Control
SBI581
igG
IP:KMT2C
P-KMT2C
KMT2C
TAOK3
KMT2C
Input
pan Phospho
-Serine/Threonine
Tubulin
B
KYSE150
DMSO
SBI581
P-KMT2C
(S4588)
P-KMT2C
(T4589)
KMT2C
TAOK3
Tubulin
C
ECA109
IC_{50}=2022 nm/l
Cell viability
Log(Concentration, nm/l)
D
KYSE150
IC_{50}=2500 nm/l
Cell viability
Log(Concentration, nm/l)
E
***
IHC Score
Normal
ESCC
F
***
IHC Score
Normal
Tumor
G
***
IHC Score
Normal
ESCC
H
***
IHC Score
Normal
Tumor

Fig. S6 The inhibitory effect of SBI-581 to TAOK3 and the IC_{50} of SBI-581 affecting ESCC cells survival at 48h are confirmed, and both TAOK3 and IRGM are overexpressed in paired or non-paired ESCC tissues of the metastasis subgroup. A CO-IP analysis of the expression level of the pan-serine/threonine phosphorylated KMT2C when SBI-581 (100nm/L) was used. B Western blot analysis of the expression of P-KMT2C (S4588) and P-KMT2C (T4589) when SBI-581 was applied in KYSE150 cells. C-D CCK8 analysis of the IC_{50} of SBI-581in terms of affecting ECA109 and KYSE150 survival at 48h. E-F IHC analysis of the expression of protein TAOK3 in paired or non-paired ESCC tissues of metastasis subgroup. G-H IHC analysis of the expression of protein IRGM in paired or non-paired ESCC tissues of metastasis subgroup.

Tables

Supplementary table 1 Primers for qPCR assays

TAOK1	forward	5'-ATGTTGGCTGTCCTGGTGTATTGC-3'
	reverse	5'-AATGCTGTTAGGAAGAAGGGTGGTG-3'
TAOK2	forward	5'-CTGCCGTAACCGAGACCACTTTG-3'
	reverse	5'-GCTGTCGTCGCATCCGCTTATAG-3'
TAOK3	forward	5'- GCTTCTCCTGCCAACTCCTTCG -3'
	reverse	5'- CCGTTCCGCCAATTCAATACAAGTG -3'
ETV5	forward	5'-CAGCACACGGGTTCCAGTCAC-3'
	reverse	5'-TGGCAGTTAGGCACTTCTGAATCG-3'
IRGM	forward	5'- AATCTTGCTGCTGCTCATTCTTTGG -3'
	reverse	5'- GCGGCGAGTCTGGAGTTGTTC -3'
IRGM-site1(for CHIP)	forward	5'-TTTTCTGATAATGATCAAACAGA-3'
	reverse	5'-CATGTGAGGTTATATATAGTTAA-3'
IRGM-site2(for CHIP)	forward	5'- ATATGCTCACTTAGTAAATTTA -3'
	reverse	5'-AGATATATTGCCAGATACC-3'
IRGM-site3(for CHIP)	forward	5'- GAACCCACCGGAAGGAAGAAACTC-3'
	reverse	5'- TGCTTATCATTGCATTCGCCAAACC -3'

Supplementary table 2 The relationship between clinical factors and TAOK3 expression

Clinicopathological factors	TAOK3 expression		
	High	Low	P-value
**Gender**			
Male	44	21	0.806
Female	9	5	
**Age(y)**			
≤60	19	7	0.428
> 60	34	19	
**Tumor size**			
≤3cm	16	14	0.042
> 3cm	37	12	
**Metastasis***			
Negtive	35	25	0.002
Positive	18	1	
**TNM stage**			
0-II	34	26	< 0.001
III-IV	19	0	

* Metastasis here includes lymph node metastasis and other organs metastasis.

Supplementary table 3 The relationship between clinical factors and IRGM expression

Clinicopathological factors	IRGM expression		
	High	Low	P-value
**Gender**			
Male	38	27	0.286
Female	6	8	
**Age(y)**			
≤60	18	8	0.090
> 60	26	27	
**Tumor size**			
≤3cm	14	16	0.206
> 3cm	30	19	
**Metastasis***			
Negtive	27	33	0.001
Positive	17	2	
**TNM stage**			
0-II	25	35	< 0.001
III-IV	19	0	

* Metastasis here includes lymph node metastasis and other organs metastasis.
